# Distinct Spatiotemporal Expression of Serine Proteases *Prss23* and *Prss35* in Periimplantation Mouse Uterus and Dispensable Function of *Prss35* in Fertility

**DOI:** 10.1371/journal.pone.0056757

**Published:** 2013-02-22

**Authors:** Honglu Diao, Shuo Xiao, Rong Li, Fei Zhao, Xiaoqin Ye

**Affiliations:** 1 Department of Physiology and Pharmacology, College of Veterinary Medicine, The University of Georgia, Athens, Georgia, United States of America; 2 Interdisciplinary Toxicology Program, The University of Georgia, Athens, Georgia, United States of America; State Key Laboratory of Reproductive Biology, Institute of Zoology, Chinese Academy of Sciences, China

## Abstract

PRSS23 and PRSS35 are homologous proteases originally identified in mouse ovaries. In the periimplantation mouse uterus, *Prss23* was highly expressed in the preimplantation gestation day 3.5 (D3.5) uterine luminal epithelium (LE). It disappeared from the postimplantation LE and reappeared in the stromal compartment next to the myometrium on D6.5. It was undetectable in the embryo from D4.5 to D6.5 but highly expressed in the embryo on D7.5. *Prss35* became detectable in the uterine stromal compartment surrounding the embryo on D4.5 and shifted towards the mesometrial side of the stromal compartment next to the embryo from D5.5 to D7.5. In the ovariectomized uterus, *Prss23* was moderately and *Prss35* was dramatically downregulated by progesterone and 17β-estradiol. Based on the expression of *Prss35* in granulosa cells and corpus luteum of the ovary and the early pregnant uterus, we hypothesized that PRSS35 might play a role in female reproduction, especially in oocyte development, ovulation, implantation, and decidualization. This hypothesis was tested in *Prss35^(−/−)^* mice, which proved otherwise. Between wild type (WT) and *Prss35^(−/−)^* mice, superovulation of immature females produced comparable numbers of cumulus-oocyte complexes; there were comparable numbers of implantation sites detected on D4.5 and D7.5; there were no obvious differences in the expression of implantation and decidualization marker genes in D4.5 or D7.5 uteri. Comparable mRNA expression levels of a few known protease-related genes in the WT and *Prss35^(−/−)^* D4.5 uteri indicated no compensatory upregulation. Comparable litter sizes from WT × WT and *Prss35* ^(−/−)^× *Prss35* ^(−/−)^ crosses suggested that *Prss35* gene was unessential for fertility and embryo development. *Prss35* gene has been linked to cleft lip/palate in humans. However, no obvious such defects were observed in *Prss35^(−/−)^* mice. This study demonstrates the distinct expression of *Prss23* and *Prss35* in the periimplantation uterus and the dispensable role of *Prss35* in fertility and embryo development.

## Introduction

Proteases (>600) are categorized into five main groups: metallo, serine, cysteine, aspartic/glutamate, and threonine based on the nature of their active-site catalytic residue. Serine proteases (PRSS) are grouped into 13 classes and 40 families characterized by the presence of serine (Ser) as the nucleophilic amino acid at the enzyme’s active site [1]–[3]. PRSS23 and PRSS35 belong to the trypsin class of serine proteases [4].

PRSS35 was originally identified as a novel mouse ovary-selective gene using suppression subtractive hybridization and PRSS23 was subsequently identified as a homologous protease of PRSS35 via BLAST search [4]. Both genes have two exons with the coding region in exon 2. Although their proteolytic activities have not yet been characterized, both possess general features of serine proteases. However, the canonical Ser is replaced by a threonine (Thr) in PRSS35 [4].

Despite their high sequence homology, *Prss35* and *Prss23* have their unique expression and regulation patterns in the mouse ovary [4], [5]. *Prss35* mRNA is localized in the theca layers of developing follicles, granulosa cells of preovulatory and ovulatory follicles, and the forming and regressing corpus luteum. *Prss23* mRNA is highly detected in the granulosa cells of the secondary/early antral follicles, it is also expressed in the ovarian stroma and theca tissues just before ovulation. *Prss35* mRNA is upregulated around the time of ovulation and remains elevated in the developing corpus luteum; while *Prss23* mRNA is transiently downregulated after ovulation induction and again in the postovulatory period. *Prss35* expression is progesterone-dependent prior to follicle rupture and upregulated by gonadotropins; while *Prss23* expression is independent of progesterone and downregulated by gonadotropins.

Dot blots of adult mouse tissues indicate that *Prss35* is detectable in the ovary only, and *Prss23* is detectable in a wide range of tissues, including a high level in the uterus [4]. A recent study shows that *Prss23* is localized in heifer’s uterine luminal epithelium (LE) and is upregulated in the heifer uterus from gestation day 7 (D7) to D13 [6], suggesting its uterine regulation during early pregnancy in heifers.

We have been studying the molecular mechanisms for the establishment of uterine receptivity using a mouse model deficient of the third lysophosphatidic acid (LPA) receptor (*Lpar3* ^(−/−)^). *Lpar3* is mainly detected in the preimplantation D3.5 LE in wild type (WT) mice and *Lpar3* ^(−/−)^ females have delayed uterine receptivity for embryo implantation [7]. Microarray analysis indicated that *Prss23* was the most differentially expressed *Prss* gene between D4.5 WT and *Lpar3* ^(−/−)^ LE cells. We analyzed the expression of both *Prss23* and its homologous protease gene *Prss35* in the periimplantation mouse uterus and found their distinct spatiotemporal expression patterns in the LE and the stromal compartment, respectively.

Proteases play an important role in proteolysis that is essential for tissue remodeling and functions of the ovary and the uterus, which go through extensive tissue remodeling during estrous cycle and pregnancy [8]–[10]. The spatiotemporal expression of *Prss23* and *Prss35* in the ovary and the uterus led us to hypothesize that PRSS23 and PRSS35 may be involved in ovarian and uterine functions [4], [5]. In this study, we tested the hypothesis on PRSS35 in *Prss35* ^(−/−)^ mice.

## Materials and Methods

### Animals and Genotyping

WT and *Lpar3* ^(−/−)^ mice (C57BL6/129svj mixed background) were from a colony at the University of Georgia, which was originally derived from a colony at The Scripps Research Institute [7]. They were genotyped as previously described [7]. *Prss35* ^(−/−)^ mice were derived from the mouse strain B6/129S5-*Prss35^tm1Lex^*/Mmucd (identification number 032535-UCD) purchased from the Mutant Mouse Regional Resource Center (MMRRC) at UC Davis, a NCRR-NIH funded strain repository. *Prss35* ^(−/−)^ mice were genotyped using tail genomic DNA and four primers: PRSS35 DNA419.18, PRSS35 DNA419.33, PRSS35 DNA419.34 and PRSS35 GT-IRES (Table 1) in PCR reactions. The PCR cycles were set as follows: 10 cycles of 94°C for 15 s, 65°C for 30 s (decreasing 1°C/cycle), and 72°C for 40 s; and 30 cycles of 94°C for 15 s, 55°C for 30 s and 72°C for 40 s. The expected PCR product sizes for WT (PRSS35 DNA419.18 and PRSS35 DNA419.33) and targeted alleles (PRSS35 DNA419.34 and PRSS35 GT-IRES) were 355 bp and 574 bp, respectively. All mice were housed in polypropylene cages with free access to regular food and water from water sip tubes in a reverse osmosis system. The animal facility is on a 12-hour light/dark cycle (7∶00 AM to 7∶00 PM) at 23±1°C with 30–50% relative humidity. All methods used in this study were approved by the University of Georgia Institutional Animal Care and Use Committee (IACUC) and conform to National Institutes of Health guidelines and public law.

**Table 1:** Primers used for realtime PCR, making probes for *in situ* hybridization, and genotyping of *Prss35* ^(−/−)^ mice.

Primer	Sequence	GenBankAccession no.	Productsize (bp)
*Prss8*	F: AGAGAACACAGCAGGGAAG	NM_133351.1	399
	R: CATGTCCTGCTGGATAGTGT		
*Prss12*	F: AGCTGCTCAGGAAAAGAAGT	NM_008939	351
	R: TGTGCACTTCACATTATCCA		
*Prss23*	F: GAAAGGGACACAGAAACTCC	NM_029614.3	372
	R: TGCTGGTAGAGAAGGTCGTA		
*Prss35*	F: GGACGGGAGGATACAGTAAG	NM_178738	347
	R: CTTTCACCCTGGTTAAGGTT		
*Isp1*	F: GCTGGCAGTGTCTGTACAAT	XM_001477507	371
	R: TGAGTCACCATAGCAGGAAT		
*Isp2*	F: ACCCTCAGTGGATTCTGACT	NM_053259	280
	R: ACAGCTCTTGTTGTCCTGAA		
*SerpinA3N*	F: CCTACTTCCGAGATGAGGAG	NM_009252.2	390
	R: GGACAAATTTGACTCCAGTG		
*SerpinG1*	F: CACTAAAGGGCTTTTCATCC	NM_009776.2	396
	R: CCTTCAAAGTATGGTCATCG		
*Spink3*	F: AGTTCTTCTGGCTTTTGCAC	NM_009258	340
	R: TTATTCAACGAACCCACTTG		
*Hprt1*	F: CAATCAAGACATTCTTTCCAGT	NM_013556	172
	R: GCTGACCTGCTGGATTACAT		
*Cox-2*	F: CTACATCCTGACCCACTTCA	NM_011198.3	387
	R: GGTCCTCGCTTATGATCTGT		
*Gja1*	F: CCTACATCATCAGCATCCTC	NM_010288.2	397
	R: TACCCAGGAGGAGACATAGG		
*Gjb2*	F: CAGCATTGGAAAGATCTGG	NM_008125.2	397
	R: GAAGACGGCTTCAAAGATG		
*Abp1*	F: TACCCTAATGGTGTGATGGA	NM_029638.1	398
	R: TCAGCCATAGAGTGGATCTG		
*Dtprp*	F: GCTCAGATCCCCTTGTGAT	NM_010088.1	396
	R: GGTCATCATGGATTTCTCTG		
*Prlpi*	F: TATGTGACTGCCACACGATA	NM_013766.1	379
	R: CCCTCCAGAACGACTTTATT		
*Gapdh*	F: GCCGAGAATGGGAAGCTTGTCAT	XM_001476707	230
	R: GTGGTTCACACCCATCACAAACAT		
PRSS35 DNA419.18	GCATCGAATGTCAGGAAGAG		355
PRSS35 DNA419.33	CTGCCTTTGACATAGTCCTTC		
PRSS35 DNA419.34	TGGAGCAGTGAACACGCAGAG		574
PRSS35 GT-IRES	CCCTAGGAATGCTCGTCAAGA		

F: forward primer; R: reverse primer. 5′→3′.

### *In situ* Hybridization

Gestation day 3.5 (D3.5) ∼ D7.5 WT uterus, D4.5 WT ovary, D3.5 and D4.5 *Lpar3* ^(−/−)^ uterus, D4.5 *Prss35* ^(−/−)^ ovary, D4.5 and D7.5 *Prss35* ^(−/−)^ uterus were snap-frozen and *in situ* hybridization was performed as previously descried [11]–[13]. Sense and antisense probes for *Prss23*, *Prss35*, *Gjb2* (gap junction protein, beta 2), *Gja1* (gap junction protein, alpha 1), *Cox-2* (cyclooxygenase 2), *Dtprp* (decidual/trophoblast prolactin-related protein), *Prlpi* (prolactin-like protein I), and *Abp1* (amiloride binding protein 1) were synthesized from cDNA fragment amplified with their respective gene specific primer pairs (Table1).

### Hormonal Treatment

Hormonal treatment on ovariectomized WT mice was done as previously described [13], [14]. Briefly, in the vehicle-treated group and the progesterone (P4)-treated group, the ovariectomized virgin C57BL6 mice (recovered for 2 weeks after surgery) were injected with 0.1 ml vehicle (oil) or P4 (2 mg in 0.1 ml oil) three times on 0 h, 24 h, and 48 h, respectively. In the 17β-estradiol (E2)-treated group, the ovariectomized mice were injected with 0.1 ml oil on 0 h and 24 h, then 100 ng E2 (in 0.1 ml oil) on 48 h. In P4+ E2-treated group, the mice were treated the same as the P4-treated group except an additional injection of 100 ng E2 on 48 h. All the mice were dissected 6 hours after the last injection. The total treatment time for P4 was 54 hours and that for E2 was 6 hours. The uteri were dissected and snap-frozen for realtime PCR.

### Realtime PCR

Realtime PCR was used to quantify the expression levels of *Prss23* and *Prss35* in the ovariectomized WT uterus upon hormonal treatment and the mRNA expression levels of a few protease-related genes in the D4.5 WT uterus and the D4.5 *Prss35* ^(−/−)^ uterus. The protease-related genes examined in the D4.5 uterus included *Prss8*, *Prss12*, *Prss23*, *Prss35*, *ISP1* (implantation serine proteinase 1), *ISP2*, *Spink3* (serine peptidase inhibitor, Kazal type 3), *SerpinA3N* (serine (or cysteine) proteinase inhibitor, clade A, member 3), and *SerpinG1* (serpin peptidase inhibitor, clade G (C1 inhibitor), member 1). The gene specific primers were listed in Table 1. Realtime PCR was done as previously described [11]–[13]. The mRNA expression levels were normalized by the expression of *GAPDH* (glyceraldehyde-3-phosphate dehydrogenase). *HPRT1* (hypoxanthine phosphoribosyltransferase 1) served as the second house-keeping gene (Table 1).

### Vaginal Opening, Superovulation, Embryo Implantation, Gestation Period, and Litter Size

The vaginas of WT, *Prss35* ^(+/−)^, and *Prss35* ^(−/−)^ females were checked daily from postnatal day 22 until vaginal opening was detected, which was recorded as the age of vaginal opening. Superovulation was done on immature 21 days old WT and *Prss35* ^(−/−)^ females. They were injected intraperitoneally with 5 IU eCG (equine chorionic gonadotropin, Sigma-Aldrich) and 48 hours later with 5 IU hCG (human chorionic gonadotropin, Sigma-Aldrich). The cumulus-oocyte complexes were collected from the oviduct 16 hours after hCG injection and counted. Embryo implantation was detected on D4.5 and D7.5 using blue dye reaction as previously described [7]. Gestation period and litter size were recorded as previously described [7].

### Statistical Analysis

Statistical analyses were done using two-tail, unequal variance Student’s t test. The significant level was set at p<0.05.

**Access to online data about Prss35^tm1Lex^** can be found at: http://www.informatics.jax.org/javawi2/servlet/WIFetch?page=alleleDetail&key=665880. From this site, click on “data” at the row of “Notes”, it will lead to the detailed information about the knockout mice. For example, “Fertility” data are under “Diagnostics”, http://mmrrc.mousebiology.org/phenotype/Genentech/PRT215N1/Diagnostics/Fertility/Level_I/PRT215N1-Diagnostics-Fertility-Level_I.html, and RT-PCR results are under “Expression” then “WT Panel”, http://mmrrc.mousebiology.org/phenotype/Genentech/PRT215N1/Expression/WT_Panel/Level_I/popups/PRT215N1-Expression-WT_Panel-imageViewer-3349.html.

## Results and Discussion

### Expression of *Prss23* and *Prss35* in Periimplantation Mouse Uterus

**Figure 1:**
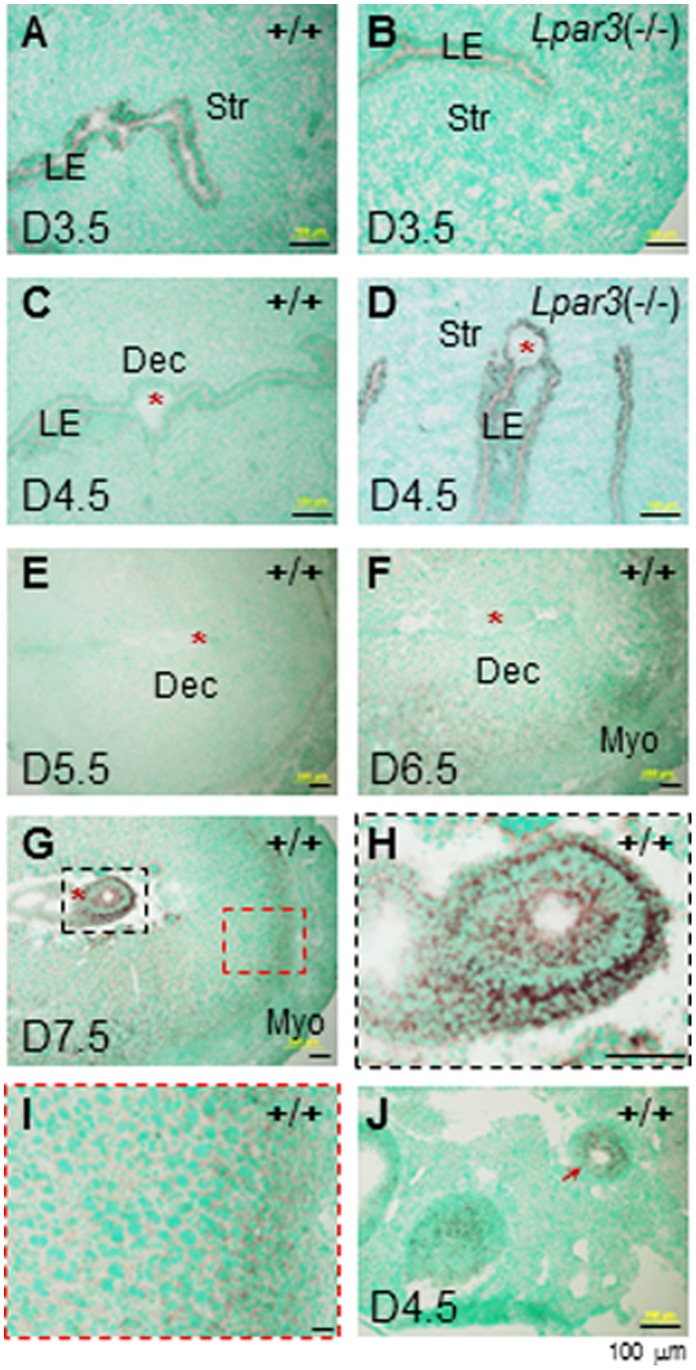
Expression of *Prss23* in the periimplantation uterus by *in situ* hybridization. A. Gestation day 3.5 (D3.5), wild-type (WT, +/+) uterus. B. D3.5 *Lpar3* ^(−/−)^ uterus. C. D4.5 WT uterus. D. D4.5 *Lpar3* ^(−/−)^ uterus. E. D5.5 WT uterus. F. D6.5 WT uterus. G. D7.5 WT uterus. H. D7.5 WT embryo enlarged from the black rectangle in G. I. D7.5 WT uterus enlarged from the red rectangle in G. J. D4.5 WT ovary as a positive control, red arrow indicating granulosa cells. *Prss23* sense probe was used as a negative control, no specific signal was detected (data not shown). Red star, embryo; LE, luminal epithelium; Str, stroma; Dec, decidual zone; Myo, myometrium; scale bar, 100 µm.

*In situ* hybridization showed expression of *Prss23* in the uterine luminal epithelium (LE) but no detectable expression in other uterine compartments of the D3.5 WT uterus (Fig. 1A). The expression level of *Prss23* was greatly decreased from LE in the D4.5 WT uterus and it was undetectable in other uterine compartments (Fig. 1C). It was detected in the LE of the D3.5 and the D4.5 *Lpar3* ^(−/−)^ uteri (Figs. 1B, 1D), which had delayed uterine receptivity for embryo implantation [7]. These data demonstrated that *Prss23* was downregulated in the LE upon embryo implantation, which normally initiates around D4.0 in WT mice [11], [15]. No significant levels of *Prss23* were detectable in the D5.5 WT uterus (Fig. 1E). It reappeared at a relatively low level in the stromal compartment next to the myometrium in the D6.5 WT uterus (Fig. 1F). It remained there in the D7.5 WT uterus and interestingly, it was highly expressed in the D7.5 embryo (Figs. 1G∼1I). Positive control indicated *Prss23* expression in the granulosa cells of the D4.5 WT ovary (Fig. 1J) [4], [5].

**Figure 2:**
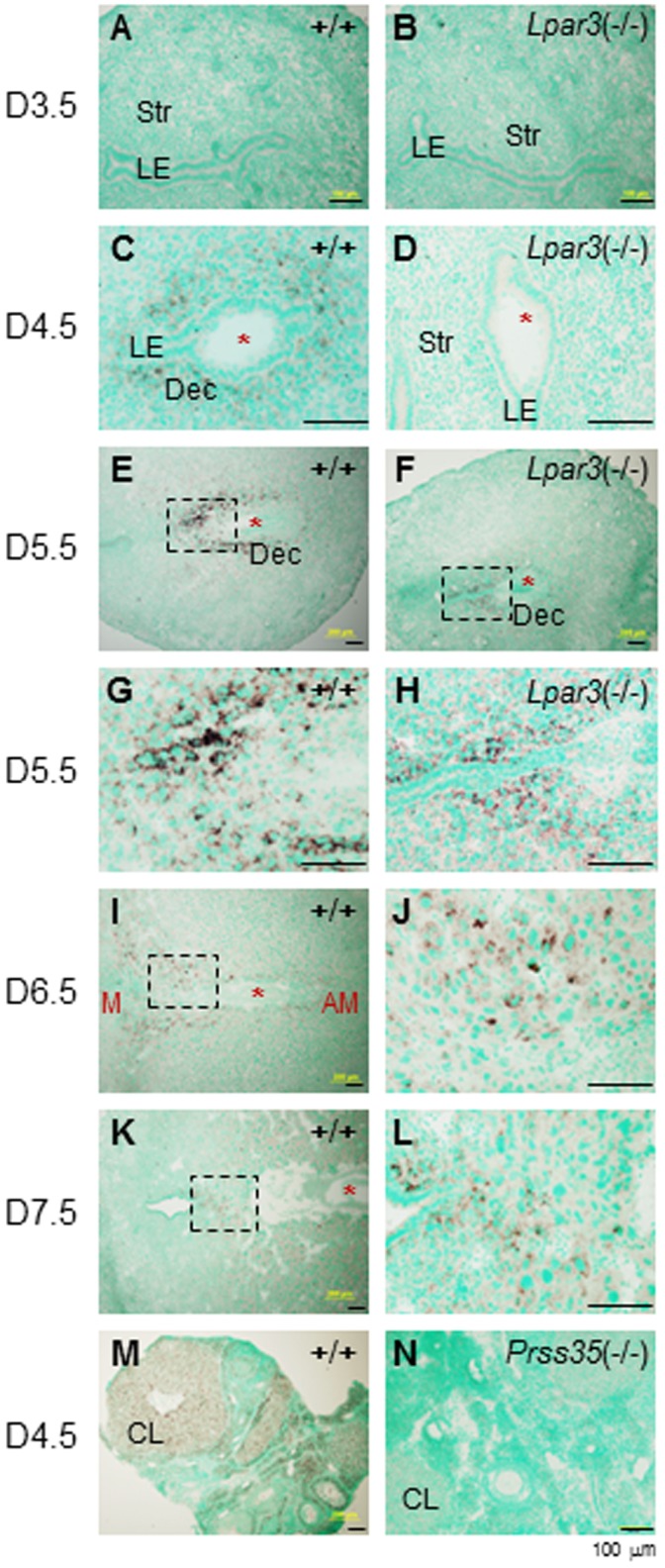
Expression of *Prss35* in the periimplantation uterus by *in situ* hybridization. A. Gestation day 3.5 (D3.5), wild-type (WT, +/+) uterus. B. D3.5 *Lpar3* ^(−/−)^ uterus. C. D4.5 WT uterus. D. D4.5 *Lpar3* ^(−/−)^ uterus. E. D5.5 WT uterus. F. D5.5 *Lpar3* ^(−/−)^ uterus. G. D5.5 WT uterus enlarged from the black rectangle in E. H. D5.5 *Lpar3* ^(−/−)^ uterus enlarged from the black rectangle in F. I. D6.5 WT uterus. M, mesometrial side; AM, antimesometrial side. J. D6.5 WT uterus enlarged from the black rectangle in I. K. D7.5 WT uterus. L. D7.5 WT uterus enlarged from the black rectangle in K. M. D4.5 WT ovary as a positive control. *Prss35* sense probe was used as a negative control, no specific signal was detected (data not shown). N. D4.5 *Prss35* ^(−/−)^ ovary. Red star, embryo; LE, luminal epithelium; Str, stroma; Dec, decidual zone; CL, corpus luteum; scale bars, 100 µm.

*Prss35* was undetectable in the D3.5 WT uterus by *in situ* hybridization (Fig. 2A). It was detected in the D4.5 WT stromal compartment surrounding the embryo (Fig. 2C). It was undetectable in the D3.5 and the D4.5 *Lpar3* ^(−/−)^ uteri (Figs. 2B, 2D), in which embryo implantation had not occurred yet [7], and it became detectable in the stromal compartment in the D5.5 *Lpar3* ^(−/−)^ uterus (Figs. 2F, 2H). Strong staining shifted to the stromal compartment on the mesometrial side of the D5.5 WT uterus (Figs. 2E, 2G). Such shifting continued on D6.5 although the intensity of expression decreased (Figs. 2I, 2J). *Prss35* remained detectable at a lower expression level in the stromal compartment on the mesometrial side of the D7.5 WT uterus (Figs. 2K, 2L), but it was undetectable in the embryo from D4.5 to D7.5 (Figs. 2C, 2D, 2E, 2F, 2I, 2K). The D4.5 WT ovary was used as a positive control for *Prss35* (Fig. 2M).

In the original study that identified PRSS35 [4], *Prss35* was only detectable in the mouse ovary but not other adult mouse tissues, including the uterus examined using dot blot. Our study indicated that *Prss35* expression was only detected in the stromal compartment upon implantation during pregnancy, which may explain why dot blot couldn’t detect *Prss35* in the non-pregnant adult mouse uterus [4]. RT-PCR data from MMRRC website (http://mmrrc.mousebiology.org/phenotype/Genentech/PRT215N1/Expression/WT_Panel/Level_I/popups/PRT215N1-Expression-WT_Panel-imageViewer-3349.html) indicate expression of *Prss35* in most tissues (no uterus) examined, including several tissues that had undetectable levels of *Prss35* by dot blot [4].

Despite their high sequence homology [4], *Prss23* and *Prss35* had no obvious overlapping spatiotemporal expression in the periimplantation mouse uterus (Figs. 1, 2), suggesting that they have potentially different roles in uterine remodeling during early pregnancy. Since *Prss23* is expressed in the LE before embryo implantation, it is possible that it could be involved in the LE preparation for the initial implantation processes, such as embryo attachment. On the other hand, *Prss35* is only detected in the stromal compartment (Fig. 2). Interestingly, the main localization of *Prss35* is on the mesometrial side of the embryo (Figs. 2E, 2G).

### Hormonal Regulation of *Prss23* and *Prss35* in Ovariectomized Mouse Uterus

**Figure 3:**
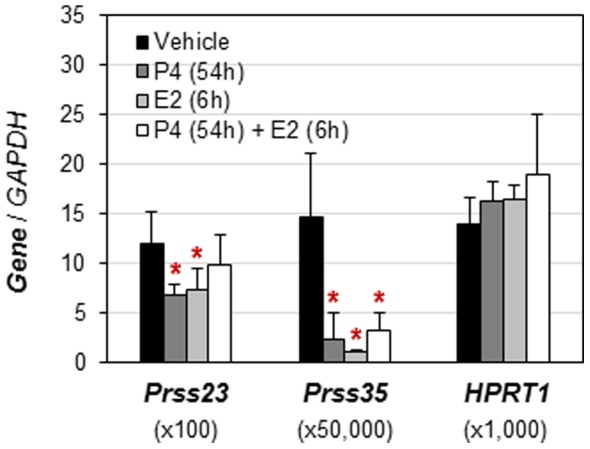
Regulation of *Prss23* and *Prss35* in ovariectomized wild-type mouse uterus upon treatments of progesterone (P4), 17β-estradiol (E2), or P4+E2. The mRNA expression levels were normalized by the expression of *GAPDH* (glyceraldehyde-3-phosphate dehydrogenase). *HPRT1* (hypoxanthine phosphoribosyltransferase 1) served as the second house-keeping gene. N = 6. Error bars, standard deviation. *p<0.05, compared to vehicle-treated group.

In the ovariectomized WT uterus, *Prss23* was moderately but significantly downregulated (<1-fold) upon P4 or E2 treatments. There was no significant difference in *Prss23* expression levels between vehicle and P4+ E2-treat groups (Fig. 3). *Prss35* was dramatically downregulated (>5-fold) upon P4 or E2 treatments. It was also significantly downregulated by P4+ E2 treatment (Fig. 3). The house-keeping gene *HPRT1* was not regulated by these hormonal treatments (Fig. 3).

Since the magnitude of uterine *Prss23* upregulation during early pregnancy is decreased in heifers with low P4 [6], it suggests that P4 might upregulate *Prss23* in the early pregnant heifer uterus. *Prss23* mRNA can be induced by E2-activated ERα in MCF-7 breast cancer cells [16]. However, *Prss23* is moderately downregulated by P4 and E2 in the ovariectomized mouse uterus under the treatment regimen (Fig. 3). These observations suggest that *Prss23* could potentially be differentially regulated by P4 in the uterus in different species and/or under different experimental settings, such as natural pregnancy [6] and ovariectomy (Fig. 3), as well as that *Prss23* could be differentially regulated by E2 under different experimental settings, such as breast cancer cells [16] and the ovariectomized mouse uterus (Fig. 3).

*Prss35* is regulated by P4 in the mouse ovary [4]. Treatment of eCG-primed mice with steroid synthesis inhibitor trilostane (TRL) reduces ovarian P4 and *Prss35* levels and the synthetic progestin R5020 could reverse the inhibitory effect of TRL on ovarian *Prss35* expression [4], indirectly indicating that P4 could upregulate *Prss35* in the mouse ovary. *Prss35* is downregulated by P4 in the ovariectomized mouse uterus (Fig. 3). These observations suggest tissue-specific regulation of *Prss35* by P4 in mice.

### General Characterization of *Prss35* ^(−/−)^ Mice

The unique spatiotemporal expression patterns of *Prss35* in the ovary [4], [5] and in the periimplantation uterus (Fig. 2) implied that PRSS35 might play roles in female reproduction. This implication was seemingly reinforced when the limited fertility data from MMRRC database indicated reduced litter sizes of 3 (normal ∼7–8) each from two *Prss35* ^(−/−)^ females mated with WT males (http://mmrrc.mousebiology.org/phenotype/Genentech/PRT215N1/Diagnostics/Fertility/Level_I/PRT215N1-Diagnostics-Fertility-Level_I.html). Therefore, we decided to study fertility in *Prss35* ^(−/−)^ females.

The deletion of *Prss35* alleles was confirmed by genotyping (data not shown) and *in situ* hybridization. *Prss35* mRNA was undetectable in the D4.5 *Prss35* ^(−/−)^ ovary (Fig. 2N) where *Prss35* was normally expressed (Fig. 2M). *Prss35* ^(−/−)^ mice were indistinguishable from their *Prss35* ^(+/−)^ or WT littermates in body weight or appearance (data not shown). The mating activities of *Prss35* ^(−/−)^ females were indistinguishable from that of the WT females (data not shown). PRSS35 has been associated with cleft lip/palate in humans [17]. However, there was no such defect observed in the *Prss35* ^(−/−)^ mice (data not shown).

### Normal Ovarian and Uterine Functions during Puberty and Pregnancy in *Prss35* ^(−/−)^ Females

**Figure 4:**
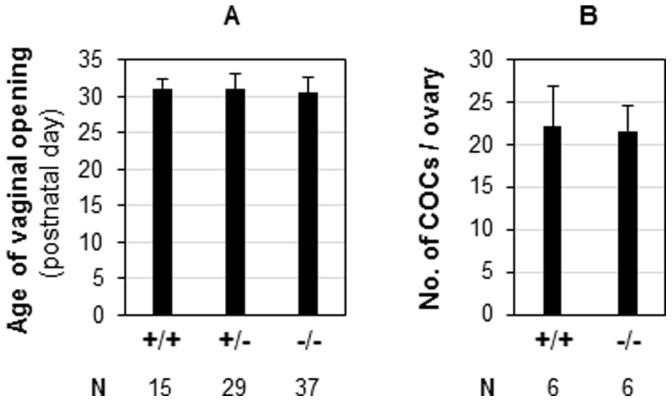
Deletion of *Prss35* on the age of vaginal opening (A) and the number of superovulated cumulus-oocyte complexes (COCs) from each ovary (B). +/+, wild-type; +/−, *Prss35* ^(+/−)^; −/−, *Prss35* ^(−/−)^; N, the number of female mice (A) or the number of ovaries (B) in each group; error bars, standard deviation.

The ovary is involved in puberty [18], [19]. Vaginal opening is an indication of puberty onset in mice [20]. The average ages of vaginal opening for WT, *Prss35* ^(+/−)^, and *Prss35* ^(−/−)^ females were comparable, around postnatal 31 days (Fig. 4A). The ovary is also involved in oocyte development and ovulation. Comparable numbers of cumulus-oocyte complexes were ovulated from superovulated immature WT and *Prss35* ^(−/−)^ females (Fig. 4B), indicating no obvious defect in oocyte development and ovulation in *Prss35* ^(−/−)^ females.

**Figure 5:**
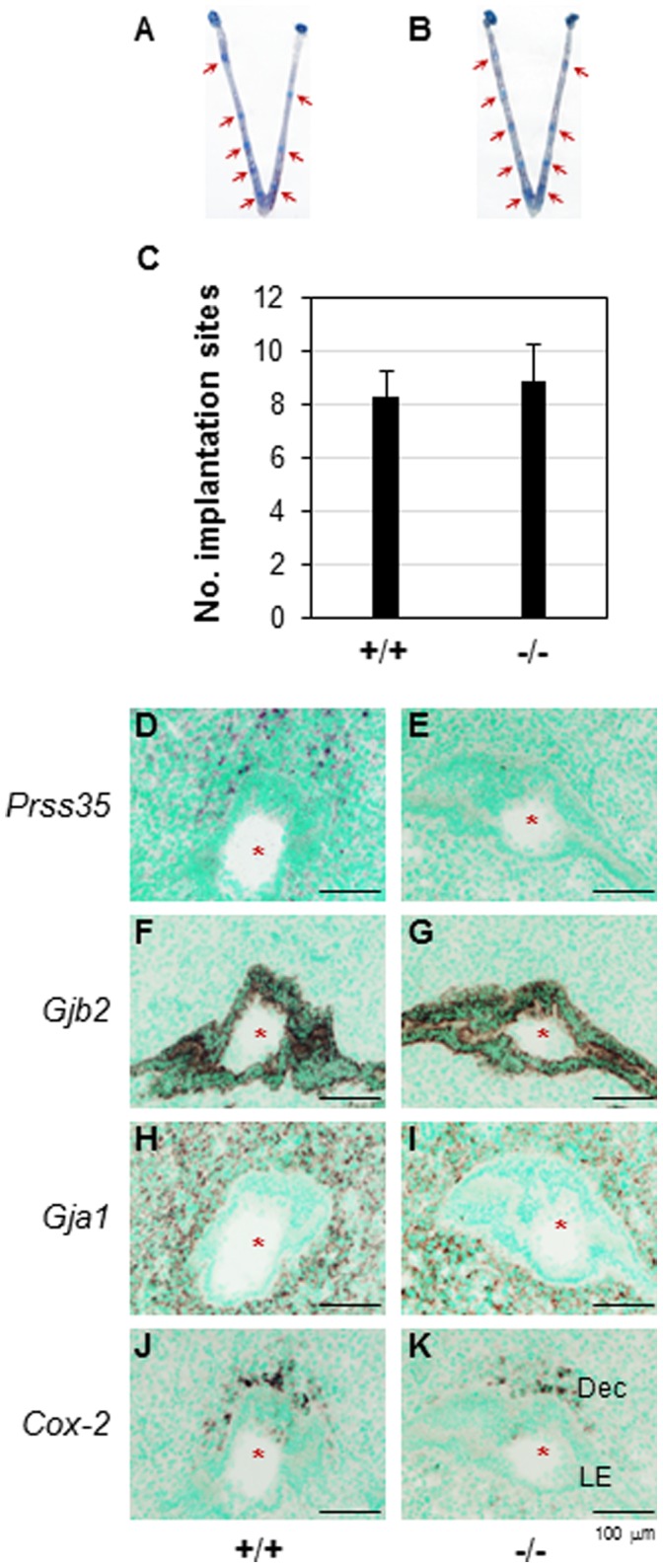
Deletion of *Prss35* on embryo implantation and the expression of implantation markers in D4.5 uterus. A. A representative uterus from D4.5 WT mice. B. A representative uterus from D4.5 *Prss35* ^(−/−)^ mice. A & B. Red arrows, implantation sites. C. The average number of implantation sites per mouse in the WT (+/+, N = 8) or *Prss35* ^(−/−)^ (−/−, N = 8) females. All the mating males were *Prss35* ^(−/−)^ for both groups except 2 WT males mated with 2 females in the WT group. Error bars, standard deviation. D∼K. Gene expression in D4.5 uterus by *in situ* hybridization. D. *Prss35* in WT uterus. E. *Prss35* in *Prss35* ^(−/−)^ uterus. F. *Gjb2* in WT uterus. G. *Gjb2* in *Prss35* ^(−/−)^ uterus. H. *Gja1* in WT uterus. I. *Gja1* in *Prss35* ^(−/−)^ uterus. J. *Cox-2* in WT uterus. K. *Cox-2* in *Prss35* ^(−/−)^ uterus. Red star, embryo; LE, luminal epithelium; Dec, decidual zone; scale bar**s**, 100 µm.

**Figure 6:**
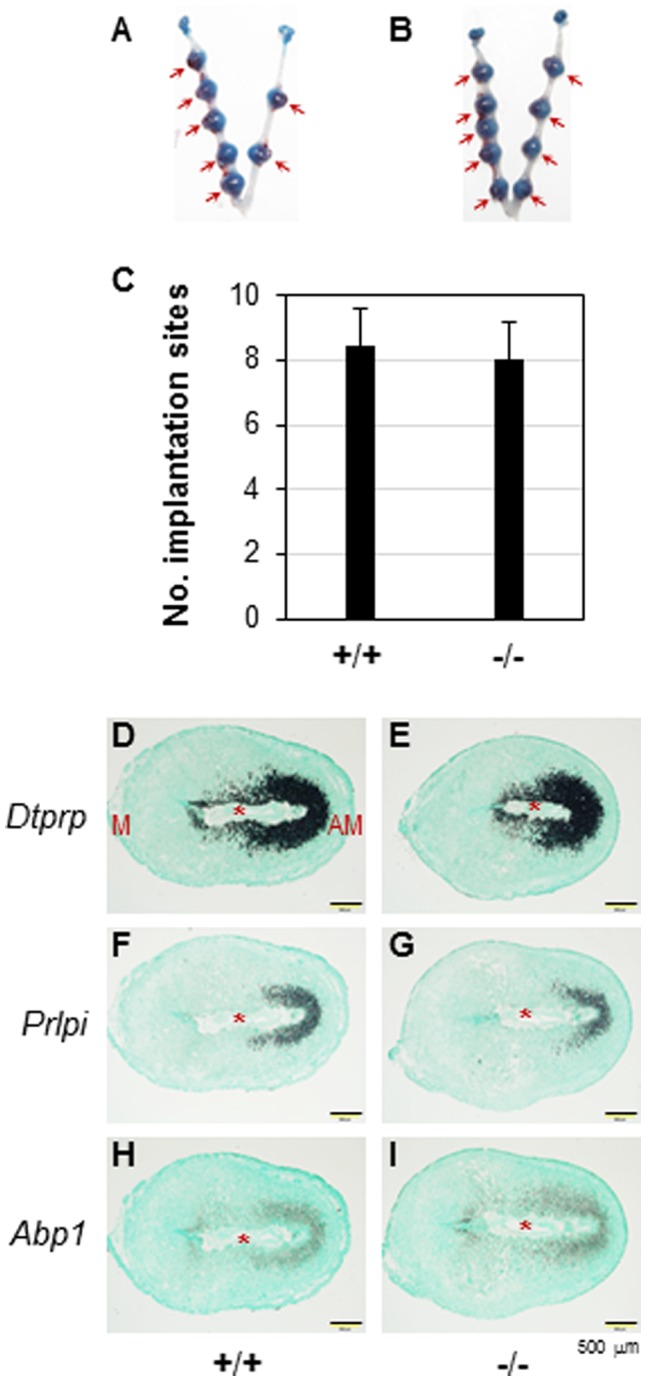
Deletion of *Prss35* on embryo implantation and the expression of decidualization markers in D7.5 uterus. A. A representative uterus from D7.5 WT mice. B. A representative uterus from D7.5 *Prss35* ^(−/−)^ mice. A & B. Red arrows, implantation sites. C. The average number of implantation sites per mouse in the WT (+/+, N = 7) or *Prss35* ^(−/−)^ (−/−, N = 7) females. All the mating males were *Prss35* ^(−/−)^ for both groups except one WT male mated with one female in the WT group. Error bars, standard deviation. D∼I. Expression of decidualization markers in D7.5 uterus by *in situ* hybridization. D. *Dtprp* in WT uterus. E. *Dtprp* in *Prss35* ^(−/−)^ uterus. F. *Prlpi* in WT uterus. G. *Prlpi* in *Prss35* ^(−/−)^ uterus. H. *Abp1* in WT uterus. I. *Abp1* in *Prss35* ^(−/−)^ uterus. Red star, embryo; M, mesometrial side; AM, antimesometrial side; scale bars, 500 µm.

The corpus luteum, in which *Prss35* is highly expressed (Fig. 2M) [4], [5], secretes P4 that is essential for establishing a receptive uterus for embryo implantation and for stimulating decidualization in the uterine stromal compartment upon embryo implantation [21]. The following data demonstrated that the corpus luteum in *Prss35* ^(−/−)^ females had normal function. First, embryo implantation, including the timing of implantation, which was based on the appearance of the blue bands that indicated the implantation sites, the spacing among the implantation sites along the uterine horns, and the average number of implantation sites detected on D4.5, was comparable between WT and age-matched *Prss35* ^(−/−)^ females (Figs. 5A∼5C). Normal embryo implantation was also confirmed at the molecular level. *In situ* hybridization confirmed the localization of *Prss35* in the D4.5 WT uterus (Fig. 5D) and the absence of *Prss35* in the D4.5 *Prss35* ^(−/−)^ uterus (Fig. 5E). It detected comparable expression of implantation markers, such as *Gjb2* (Figs. 5F, 5G) [22], *Gja1* (Figs. 5H, 5I) [22], and *Cox-2* (Figs. 5J, 5K) [23], between the D4.5 WT uterus and the D4.5 *Prss35* ^(−/−)^ uterus. Second, decidualization was well developed in the D7.5 *Prss35* ^(−/−)^ uterus, which had comparable sizes and numbers of implantation sites compared to those in the D7.5 WT uterus (Figs. 6A∼6C). Normal decidualization in the D7.5 *Prss35* ^(−/−)^ uterus was also confirmed by the comparable expression of decidualization markers, such as *Dtprp* (Figs. 6D, 6E) [24], *Prlpi* (Figs. 6F, 6G) [24], and *Abp1* (Figs. 6H, 6I) [25]. Interestingly, these decidualization markers were mainly localized in the antimesometrial side (Figs. 6D–6I), while *Prss35* was mainly localized in the mesometrial side (Figs. 2E, 2I, 2K). These data also demonstrated that the *Prss35* ^(−/−)^ uterus functioned normally during early pregnancy. In addition, all the females, regardless of genotypes, had gestation periods of ∼19.5 days. Therefore, PRSS35 is not essential for ovarian and uterine functions.

### Non-essential Role of PRSS35 in Embryo Development

**Figure 7:**
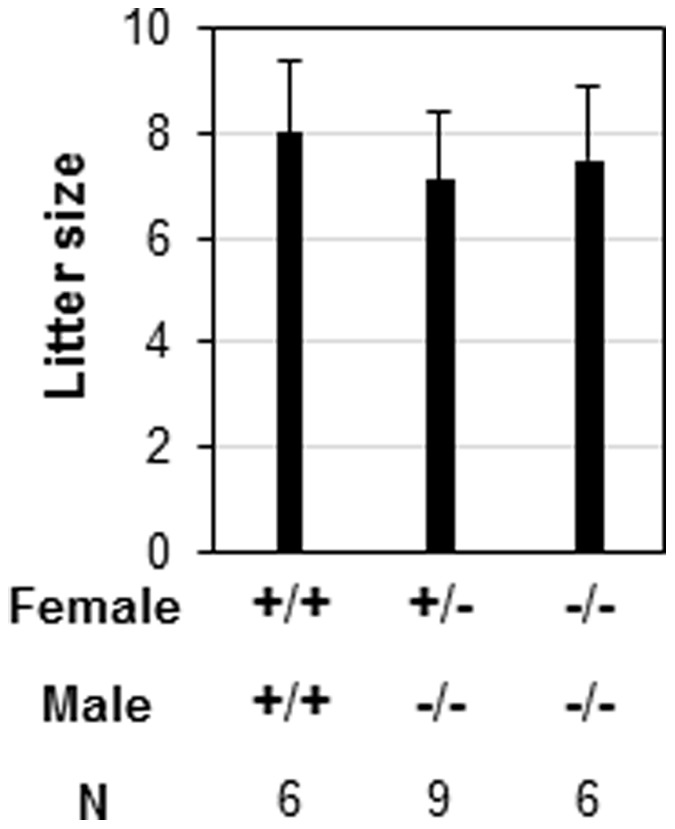
Deletion of *Prss35* on litter size. +/+, wild-type; +/−, *Prss35* ^(+/−)^; −/−, *Prss35* ^(−/−)^; N, the number of female mice in each group. Error bars, standard deviation.

When *Prss35* ^(−/−)^ males were mated with *Prss35* ^(+/−)^ or *Prss35* ^(−/−)^ females, the average litter sizes were comparable to that from WT × WT crosses (Fig. 7), indicating that PRSS35 did not play a critical role in embryo development.

### No Compensatory Upregulation of the mRNA Levels of a Few Other Protease-related Genes in the D4.5 *Prss35* ^(−/−)^ Uterus

**Figure 8:**
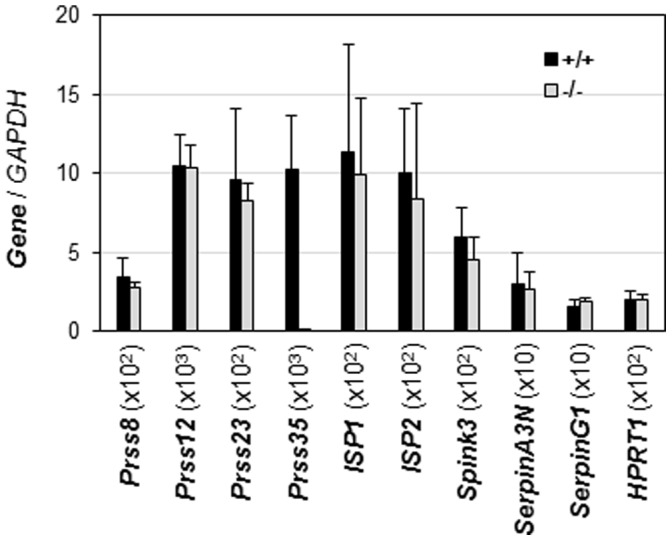
Deletion of *Prss35* on the expression of a few protease-related genes in D4.5 uterus. +/+, wild-type; −/−, *Prss35* ^(−/−)^. X-axis indicated the names of different genes. Y-axis showed the normalized mRNA expression levels by *GAPDH* x the number in the parentheses following each gene on X-axis in order to present the relative expression levels of these genes in the same figure. *Prss35* was included to confirm that *Prss35* was deleted in the *Prss35* ^(−/−)^ uterus. N = 6 (+/+) and N = 4 (−/−). Error bars, standard deviation.

Compensatory upregulation of other proteases has been reported in the uterus upon deletion of one protease, such as in the case of upregulation of matrix metalloproteinase-3 (MMP-3) and MMP-10 in the MMP-7-deficient uterus [26]. To determine if deletion of *Prss35* could lead to compensatory upregulation of other proteases in the uterus, several known uterine expressed protease-related genes were examined in D4.5 WT and *Prss35* ^(−/−)^ uteri. These genes included *Prss8* [27], *Prss12* [28], *Prss23* [4], *ISP1* [29], *ISP2* [30], *Spink3* [31], *SerpinA3N* [32], and *SerpinG1* [33]. Since *Prss35* was upregulated in the D4.5 WT uterus upon implantation (Fig. 2C), the expression levels of these genes were examined in the D4.5 WT and *Prss35* ^(−/−)^ uteri. Comparable mRNA expression levels of these protease-related genes between D4.5 WT and *Prss35* ^(−/−)^ uteri indicated no compensatory upregulation of these genes in the *Prss35* ^(−/−)^ uterus (Fig. 8). However, it could not rule out the possible compensatory upregulation of other protease-related genes in the uterus or the possible compensatory upregulation of protease-related genes in other tissues.

In summary, this study demonstrates the distinct spatiotemporal expression patterns of *Prss23* and *Prss35* in the periimplantation mouse uterus as well as the non-essential role of *Prss35* in ovarian function, uterine function, and embryo development using the *Prss35* ^(−/−)^ mouse model.
